# Attachment ability and mating behaviour of the black soldier fly *Hermetia illucens* (Diptera: Stratiomyidae)

**DOI:** 10.1007/s00359-026-01815-3

**Published:** 2026-05-17

**Authors:** Manuela Rebora, Giulia Petroni, Valerio Saitta, Silvana Piersanti, Gianandrea Salerno, Stanislav Gorb

**Affiliations:** 1 Dipartimento di Chimica, Biologia e Biotecnologie, University of Perugia, via Elce di Sotto 8, 06123 Perugia, Italy; 2 Dipartimento di Scienze Agrarie, Alimentari e Ambientali, University of Perugia, Borgo XX Giugno 74, 06121 Perugia, Italy; 3 Department of Functional Morphology and Biomechanics, Zoological Institute, Kiel University, Am Botanischen Garten 9, 24098 Kiel, Germany

**Keywords:** Tarsi, Sexual dimorphism, Reproduction, Adhesive setae, Biomechanics

## Abstract

*Hermetia illucens* (Diptera: Stratiomyidae), also known as black soldier fly (BSF), offers an important sustainable approach to waste management and resource recovery, since its larvae can reduce organic waste volumes, transform them into biomass, and generate protein-rich feed, biodiesel precursors, and organic fertilizers. Despite the abundance of information on its larval stage, the reproductive biology of the adult is still poorly understood. The present study, through behavioural tests, cryo-scanning electron microscopy analysis and centrifugal force tester experiments, deepens the knowledge on the mechanical interaction between male tarsi and the body of conspecifics during mating and competition, describes the sexual dimorphism in BSF tarsal attachment devices and provides information on the attachment ability of both sexes to artificial surfaces with different features in terms of roughness and wettability. Further studies comparing adult behaviour in rearing conditions and in the wild are necessary to optimise BSF mass-rearing facilities.

## Introduction

*Hermetia illucens* L. (Diptera: Stratiomyidae), also known as the black soldier fly (BSF), is considered the “crown jewel” of the insect-based feed industry (Tomberlin and Van Huis 2020). BSF plays a significant role at the larval stage in the circular economy due to its ability to convert organic waste into valuable products for energy, food, feed, and agricultural applications (Wang and Shelomi 2017; Schmitt de Vries 2020; Mohan et al. 2023). Over the past two decades, extensive research has been conducted on larval development and the biomass produced. However, basic research concerning adult behaviour (sexual dimorphism, intra- and intersexual selection, mating and oviposition) and adult functional morphology is still scanty (Meneguz et al. 2023; Bruno et al. 2025). Apart from few behavioural studies (Tomberlin and Sheppard 2001; Giunti et al. 2018; Julita et al. 2020) in the wild or in controlled conditions reporting some data about mating behaviour, oviposition and male competition, little is known about adult biology and we can infer that adult reproduction remains one of the least understood aspects of the BSF biology (Lemke et al. 2023). After emergence, males search for and arrive at the mating site first, far away from the pupation site, where they establish territories (Tomberlin and Sheppard 2001). Such “lekking behaviour” of the BSF in the wild deserves further investigations, since male dominance and female choice are still unclear in different species belonging to the genus *Hermetia*, both in the wild and in rearing conditions (Alcock 1990, 1993; Lemke 2025), with potential differences in the two conditions. According to Tomberlin and Sheppard (2001) (observations in the wild), about two days later, females arrive to mate. Courtship consists of the male grasping the female and pressing her to the ground, mounting the dorsal side of the female body, tapping and grooming, genital contact (in rearing conditions, these last behaviours are also recognisable during competition between males (Giunti et al. 2018)), followed by mating. During courtship and mating, the male performs active wing fanning. Adults typically mate end-to-end, with pairs persisting approximately 30 min (Giunti et al. 2018; Julita et al. 2020; review in Lemke et al. 2023). After mating, females search for a suitable substrate to oviposit.

It could be important to clarify in detail intra- and intersexual selection, male and female interactions during courtship and mating, as well as the sensory cues involved in partner recognition from a distance and at close range. Likewise in other Diptera (e.g. Billeter and Levine 2013), it is assumed that, also in BSF, a multimodal communication allows partner/competitor recognition and mating/competitive behaviour, with a combination of visual, acoustic, chemical and chemotactile cues (Giunti et al. 2018). Wing sexual dimorphism due to iridescent structural colouration is particularly evident regarding the strong emission of the blue colour in BSF female wings, which induces a strong motivation to mate in males (Rebora et al. 2024). We cannot exclude a role of chemosensory cues, which can be potentially perceived by antennal sensilla from a certain distance (Scieuzo et al. 2021; Piersanti et al. 2024, 2026); electrophysiological investigations on adult antennal sensilla and behavioural tests are in progress to clarify this point.

Since males follow flying females and contact them with their legs to grasp the females to the ground, we hypothesize a good attachment ability of males to the female body surface. Moreover, after landing on the female, males keep all their tarsi in contact with the female body and touch the female body repeatedly (Giunti et al. 2018; Julita et al. 2020). In this context, the aims of the present study were as follows. (1) To describe in detail the different steps of the mating behaviour and the mechanical interaction between male tarsi and the body of conspecifics during mating and competition. (2) To describe the tarsal attachment devices of males and females and to measure the attachment ability of both sexes to artificial surfaces with different features in terms of roughness and wettability. In this regard, the wettability of the BSF body surface has been characterised using contact angle measurements.

In order to shed light on these topics, we performed: (i) behavioural observations in controlled conditions; (ii) cryo-scanning electron microscopy analysis (cryo-SEM) on male and female tarsal attachment devices and abdomen; (iii) centrifugal force tester experiments on both sexes.

Since industrial producers rely on adult flies’ reproduction, to generate insect biomass for feed, the results of the present research, shedding light on the BSF mating behaviour and attachment ability, can help to optimize large-scale industrial production of the BSF. In addition, this study can highlight special sexually dimorphic traits evolved in Diptera, in order to improve adhesion during mating, a field so far investigated mainly in representatives of Coleoptera.

## Materials and methods

### Insects

BSF pupae obtained from BugsLife srl. (Via Cantone 5, 06031 Bevagna (PG), Italy) were placed inside the rearing cage (50 × 50 × 50 cm) consisting of a wooden structure with a transparent Plexiglas lid until adult emergence. After emerging, adults were housed together in controlled condition at a temperature of 28 ± 3 °C, and relative humidity (RH%) of 60 ± 10% under a 14 L: 20 D photoperiod. To stimulate mating, a BSF breeding lamp (model BSF-4–100 J-3030 H, SPR AG Tech, China) was used, and the internal walls of the cage were lined with aluminium foil to increase light refraction. Adults were provided with water and crystallized sucrose, while *Vicia faba* var. minor (Fabaceae) seedlings were included in the rearing cage to help maintain high RH%. For oviposition, plastic boxes (7 cm in diameter, 8 cm high) filled with the Gainesville diet (a mixture of 50% wheat bran, 30% alfalfa meal, and 20% corn by weight) diluted with water were given to females. The lids of these boxes had perforations, and squares of cardboard were placed inside to serve as oviposition sites for the females. These boxes were inspected daily, and any cardboard with freshly laid eggs was removed and transferred to other containers with damp paper to retain moisture for egg hatching. After three days, the newly hatched first-instar larvae were collected using a small brush and transferred to transparent plastic containers (16 × 14 × 5 cm) with mesh lids, containing Gainesville diet mixed with water (125 cc of water per 100 g of diet) for larval growth. The pupae were then returned to the aforementioned wooden and Plexiglas cage for adult emergence. Only adults that emerged within five days were used for this study. To obtain virgin insects for behavioural experiments, adults were sexed immediately after emergence and were kept in separate cages for 2–4 days prior to use.

### Behavioural observations

The behavioural experiments were conducted in the same room used for rearing *H. illucens* under the BSF breeding lamp. Two different sets of behavioural assays were performed; each was conducted in a different cage and recorded with different video equipment. The first set aimed to analyse the initial steps of the interaction between the sexes and to construct an ethogram, whereas the second was designed to investigate in detail the mechanical interaction between the male tarsi and the female body. In both experiments, individuals selected for testing were virgin males and females 2–4 days old. To select motivated adults ready to mate, we placed the two cages with virgin males and females under the BSF breeding lamp and selected the females and males more active (flying females and males performing wing fanning).

In the first experiment, five females and one male were introduced in a transparent Plexiglas cage (45.5 × 24.5 × 35.0 cm) with one of the lateral sides constituted of a sheet of filter paper perforated, in order to introduce insects. Insects were introduced in the cage with a Falcon™ 15 ml conical centrifuge tube. Likewise, in other previous behavioural investigations on *Hermetia* species (e.g. Alcock 1990), to facilitate the visualization of males, they were marked on the dorsal side of the thorax with white nontoxic, water-based acrylic pens (UNI Posca, 0.7 mm, PC-1MR, Mitsubishi Pencil Co., Ltd., Japan). Video recording began immediately after all insects were placed in the experimental setup using an iPhone 13 Pro camera in slow-motion recording mode (240 fps) positioned directly above the cage. Nine video sequences (out of 22 obtained) had sufficient quality to allow subsequent analyses. For the video analysis, the following behaviours were recorded using Solomon Coder software: Male stationary (Stay), Male beginning to flight (Fly), Male following a female (Follow), Male attempting to grasp a female to the ground (Gra), Male attempting to mount a female (M), Successful copulation (Copula). During the video observations, the condition of the female during the behaviours performed by the focal male was recorded as follows: female stationary (Stay), at least one female flying (Fly). A flow chart of interactions between focal male and females was built by considering the frequency of transitions from one behaviour to the subsequent one. Transitions with a frequency lower than 3% were disregarded.

In the second experiment, five females and two males were initially introduced in glass cylinder covered with a Petri dish lid (9 cm in diameter x 6 cm in height). Video recordings were captured using a stereomicroscope (Optika SLX-5, Optika s.r.l., Italy) equipped with a Koppace KP-2100 camera. To record the mechanical interaction between the male tarsi and the female/male body, 20 interactions male/female and male/male were registered and analysed using Photron software to get images showing the position of the insect tarsi over the conspecific body.

### Light microscopy

Tarsal footprints of both males and females walking on glass were observed with reflection interference contrast microscopy (RICM) using an inverted bright-field microscope ZEISS Axio ObserverA1 (Carl Zeiss Microscopy GmbH) and recorded with a Sony 3CCD video camera.

The pretarsal attachment devices (pulvilli and empodium) of 11 males and 12 females were mounted in glycerine, observed and photographed with a microscope Leica DMLB connected to a Koppace FHD Camera V 2.0. The surface of empodium and of the right pulvillum of forelegs, midlegs and hindlegs of males and females were measured using the software ImageJ (Sun-Java, USA).

### Cryo-scanning Electron Microscopy (cryo-SEM)

For cryo-SEM, the shock-frozen tarsi and body of *H. illucens* (3 males and 3 females) were studied in SEM Hitachi S-4800 (Hitachi High-Technologies Corp., Tokyo, Japan) equipped with a Gatan ALTO 2500 cryo-preparation system (Gatan Inc., Abingdon, UK). Insect tarsi were sputter-coated in frozen conditions with gold-palladium (thickness: 10 nm) and examined at 3 kV acceleration voltage and temperature of − 120 °C at the cryo-stage within the microscope.

### Friction force experiments

The experiments were performed using a centrifugal force tester. The primary reason we focused on friction force rather than adhesive force is that, in our system, locomotion stability is predominantly governed by shear (frictional) interactions rather than normal pull-off forces. In legged climbing, especially under dynamic conditions, friction plays a critical role in load sharing, preventing slip, and enabling controlled detachment during gait transitions. In contrast, adhesive (normal) force measurements alone would not fully capture the functional performance of the foot during locomotion, as attachment in our system relies on the synergistic interaction between friction and adhesion. Moreover, the centrifuge setup used in this study is particularly well-suited for quantifying shear forces under controlled loading conditions, while reliable measurement of pure adhesive forces would require modifications to decouple normal and tangential components.

Before the force measurements, adults of *H. illucens* were weighted on a micro-balance (Mettler Toledo AG 204 Delta Range, Greifensee, Switzerland). Experimental insects were anaesthetized with carbon dioxide for 60 s and were rendered incapable of flight by carefully cutting their wings. Before starting experiments, insects were left to recover for 30 min. All the experiments were performed during the daytime at 22 ± 1 °C temperature and 60 ± 10% RH.

The centrifugal force tester (Gorb et al. 2001) consists of a metal drum covered with a substrate disc to be tested. The metal drum is driven by a computer-controlled motor. Just above the disc, the fibre-optic sensor monitored by a computer is mounted. After the positioning of the insect on the horizontal disc, the centrifuge drum was allowed to begin the rotation at a speed of 50 rev·min^–1^ (0.833 rev∙s–1). The position of the insect on the drum was monitored by using a combination of the focused light beam and the fibre-optic sensor. The drum speed was continuously increased until the insect lost its hold on the surface under centrifugal force. The rotational speed at contact loss, position of the insect on the drum (radius of rotation), and the insect mass were used to calculate the frictional component of the attachment force at equilibrium and the safety factor (friction force normalized by the insect body weight). Ten males and ten females (five repetitions with each fly) were tested to evaluate the insect attachment ability on artificial substrates with different roughness (smooth, 0.3, 1, 3, 9, 12 μm). Ten males and 12 females (five repetitions with each fly) were used to evaluate the insect attachment ability to hydrophobic glass, 14 males and 14 females (five repetitions with each fly) were used to evaluate the insect attachment ability to hydrophilic glass.

### Substrate preparation and characterization

Artificial substrates with different roughness made of the same material (epoxy resin) were prepared. Such epoxy resin casts were made of the templates of a clean glass surface (smooth) and polishing papers with defined asperity sizes (0.3, 1, 3, 9, 12 μm) (by using a two-step moulding method (Gorb 2007). The roughness of the substrates has been previously characterized in Salerno et al. (2018).

To prepare hydrophobic glass, the same method reported in Salerno et al. (2020) has been used. The wettability of surfaces used in experiments (hydrophilic glass, CA = 22.9 ± 1.7° and hydrophobic glass, CA = 111.9 ± 0.5°) and of the dorsal surface of BSF females was characterized by determining the contact angles of water (aqua millipore, droplet size = 1 µl, sessile drop method) using a high-speed optical contact angle measuring instrument OCAH 200 (Dataphysics Instruments GmbH, Filderstadt, Germany). Ten measurements (*n* = 10) were performed for each substrate.

### Statistical analysis

Friction force and safety factor data obtained from *H. illucens* with the centrifuge force tester on surfaces with different asperity sizes, and wettability were analysed separately for males and females with one-way analysis of variance (ANOVA) (Statistica 6.0, Statsoft Inc. 2001). Tukey’s HSD post hoc test for multiple comparisons between means was used. Data obtained from males and females were compared using a Student’s *t-*test for independent samples. The force and the safety factor per unit area of contact between females and males were compared using the Mann-Whitney Rank Sum Test.

The surface area of empodium and pulvilli of forelegs, midlegs and hindlegs of males was compared to those of females using a Student’s *t-*test for independent samples.

## Results

### Mating behaviour and interaction between male tarsi and the female body

**Fig. 1:**
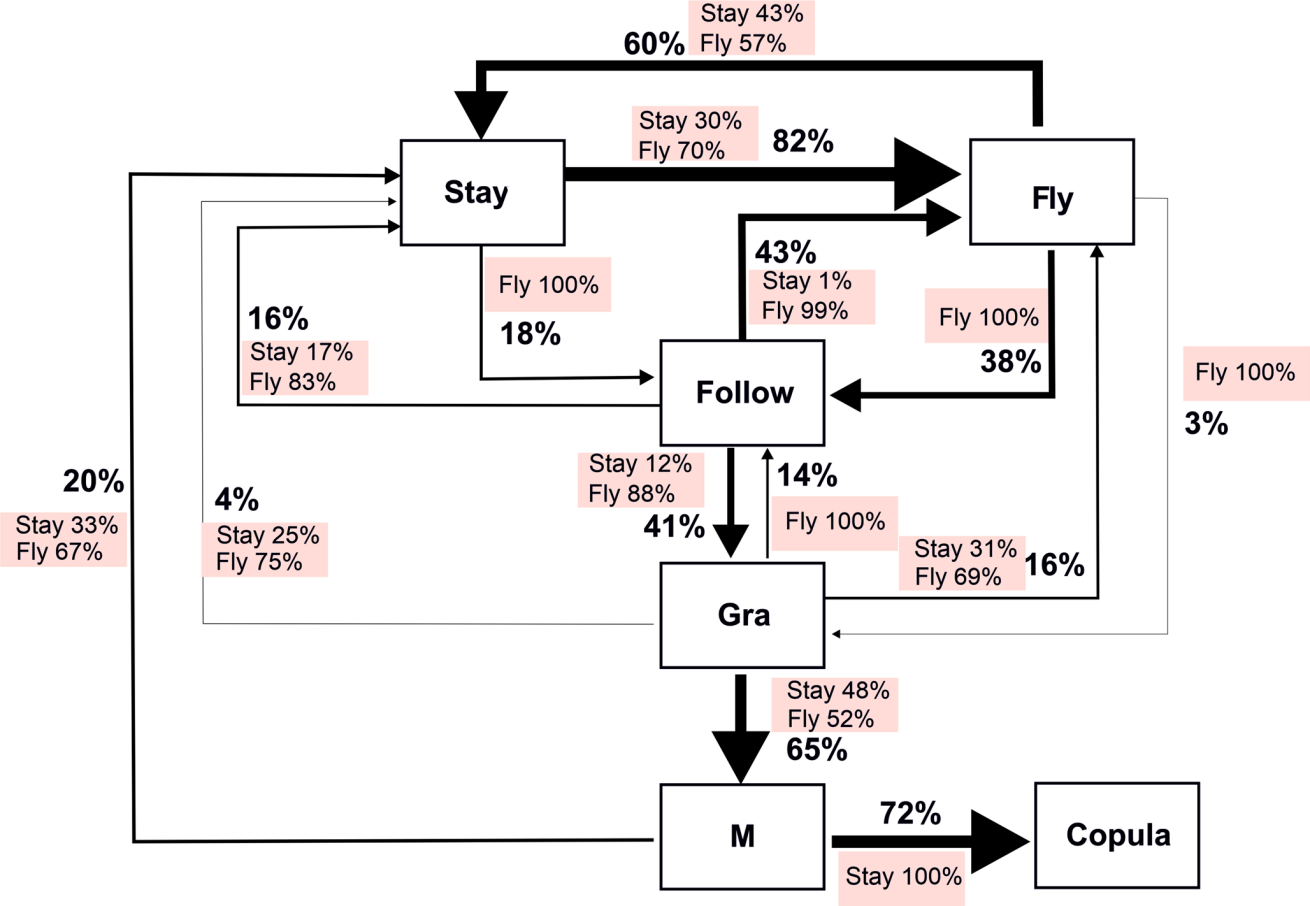
Flow chart of interactions between focal male and females of *Hermetia illucens* during mating behaviour. The following male behaviours (white boxes) were recorded: Male stationary (Stay), Male beginning to flight (Fly), Male following a flying female (Follow), Male attempting to grasp a female to the ground (Gra), Male attempting to mount a female (M), Successful copulation (Copula). The pink boxes show the condition of the females during the behaviours performed by the focal male: female stationary (Stay), at least one female flying (Fly). The size of the arrows is proportional to the number of times a behaviour was observed. Percentages represent the frequency of transition from one behaviour to the next. Transitions with a frequency lower than 3% were excluded

The typical mating interaction between male and female begins with the male staying or flying and beginning to follow the flying female (Fig. 1). In 18% of cases, the male starts to fly (from the stay condition) to follow a female (Follow). In the remaining 82% of cases, the male takes off without directly pursuing a female (Fly). Even in this latter condition, male flight was associated in 70% of cases with the simultaneous flight of at least one female, and only in 30% of cases it occurred, when all females were stationary (pink boxes in Fig. 1). From the flying state (Fly), the male returned to rest (Stay) in 60% of cases, followed a female (Follow) in 38%, or grasped a female with his legs (Gra) in 3% of cases. When the male performs grasping behaviours (Gra), the female is always in flight. From the grasping state (Gra), the male attempted mounting (M) in 65% of cases, returned to rest (Stay) in 4%, resumed flying (Fly) in 16%, or followed a female (Follow) in 14%. From mounting (M), the copula occurred in 72% of cases, whereas in 20% of cases, the male returned to a resting phase (Stay).

**Fig. 2:**
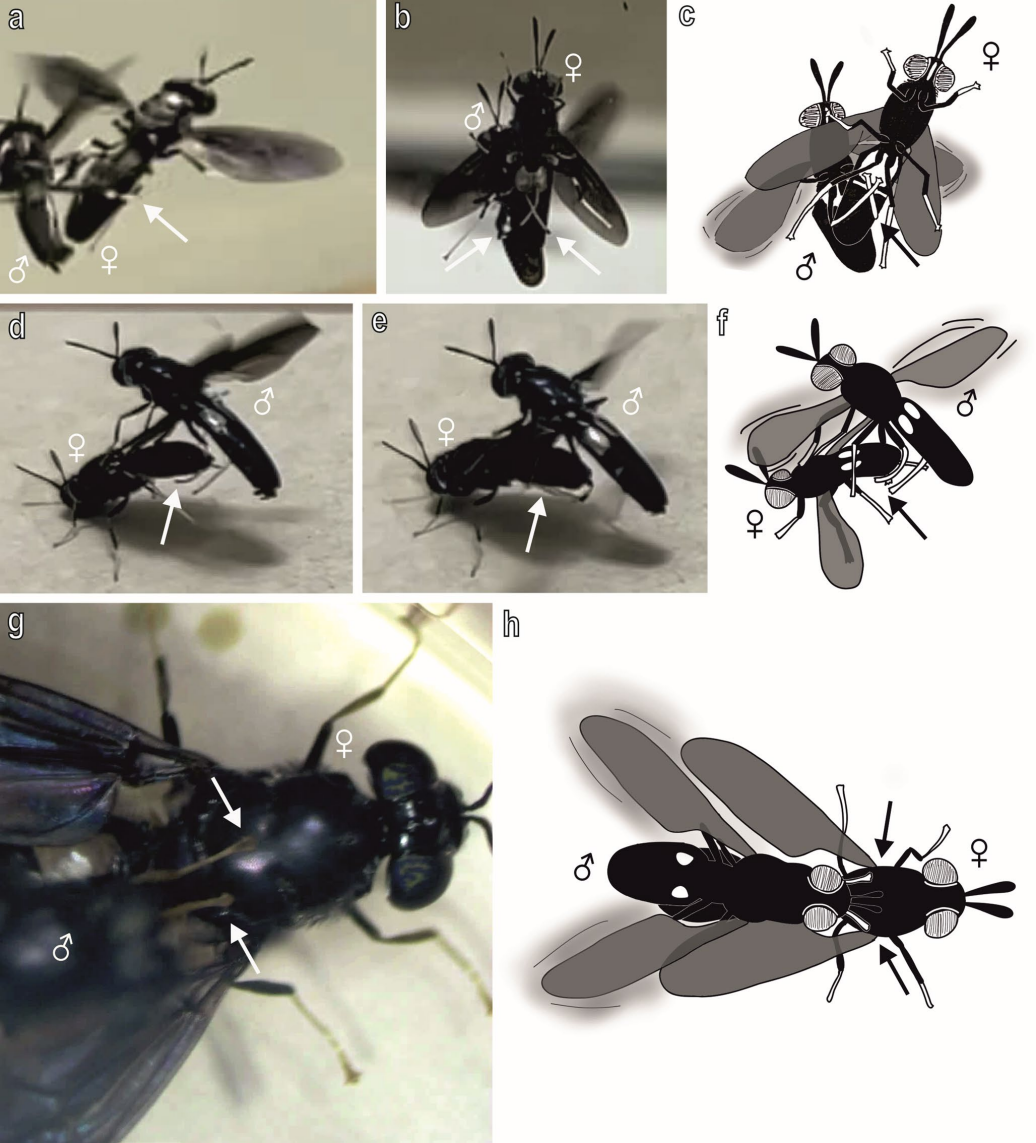
Details of the mechanical interactions (pictures in **a**, **b**, **d**, **e**, **g** and corresponding drawings in **c**, **f**, **h** between male tarsi and female body of *Hermetia illucens* during mating. **a** Male grasping the female in flight crossing its hind legs (arrow) around the female abdomen; **b** Detail showing the position of the male hindlegs crossed around the female abdomen when the couple is landing on the ground observed from the ventral side through the Plexiglas cage; **c** Schematic reconstruction of the behaviour represented in (**b**); **d**, **e** Two consecutive frames from the videorecording showing the tapping behaviour performed by the male hindlegs (arrow) on the female ventral abdomen; **f** Schematic reconstruction of the behaviour represented in (**d**); **g** Rubbing behaviour performed by the male forelegs (arrows) on the female thorax; (**h**) Schematic reconstruction of the behaviour represented in (**g**)

**Fig. 3:**
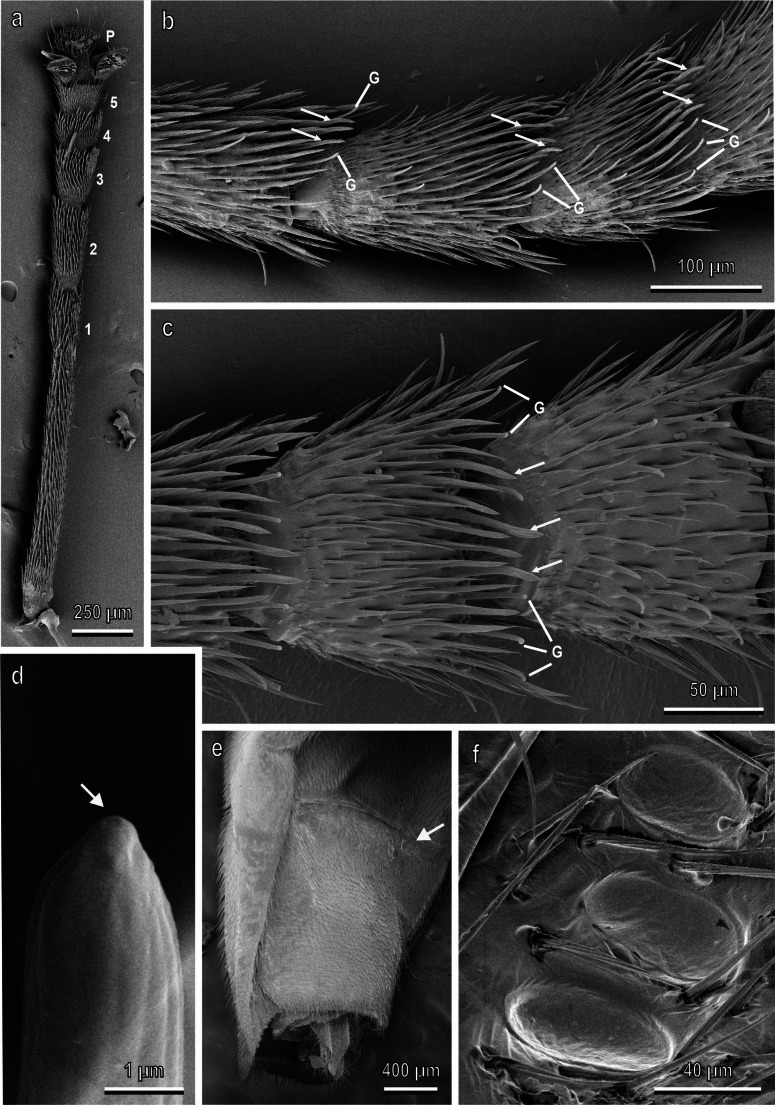
Tarsi (**a**–**d**) and female abdomen (**e**, **f**) of *Hermetia illucens* observed in cryo-SEM. **a** Ventral view of a male tarsus consisting of five segments (1–5) and the pretarsus (P); **b**, **c** Lateral view of the male tarsi (**b**) and ventral view of the female tarsi (**c**) showing the lanceolate tenent setae (arrows) located on the distal portion of each tarsal segment close to gustatory sensilla (G) (; **d** Detail of the apical pore (arrow) of a gustatory sensillum; **e** Ventral view of the hairy female abdomen with campaniform sensilla (arrow); **f** Campaniform sensilla scattered on the ventral surface of the abdomen

To grasp a female in flight and maintain its body in contact with the female body, the male typically crosses its hind legs around the female abdomen (Fig. 2a), a behaviour which is kept when the couple is landing to the ground (Fig. 2b and schematic drawing in Fig. 2c). Once landed to the ground, the male keeps all its legs in contact with the female body and exhibits tapping movements with its hind legs (Fig. 2d, e and scheme in Fig. 2f), most probably targeting the region covered by mechanoreceptors on the female abdomen (Fig. 3e, f). Once the male has achieved a balanced posture using its crossed hind legs, it performs rubbing behaviour with its foreleg tarsi on the female thorax (Fig. 2g and scheme in Fig. 2h) and the anterior abdomen; in some cases it extends to the wings. Successful genital contact occurs only when the female is receptive. In agreement with the observations by Giunti et al. (2018), receptive females do not walk or fly but slightly spread their wings, presumably to facilitate the male’s grasp and improve genital contact during copulation. Conversely, unreceptive females keep their wings tightly closed and walk or fly away. In these cases, females actively use their hind legs and walk to dislodge the male’s grip and prevent subsequent copulation. During male–female interactions, wing fanning is exhibited exclusively by the male during grasping, mating and copulation.

In the second experiment, agonistic encounters between males are characterized by a pattern of interactions similar to those observed during mating attempts. One male grasps another male during flight using its crossed hind legs around its abdomen to immobilize the other male. Tapping movements of the hind legs are also observed during male–male interactions, targeting the abdominal region of the opponent. As in mating contexts, foreleg rubbing behaviours are sometimes performed on the lower thorax and anterior abdomen of the opponent male. A distinctive feature of male–male interactions is the simultaneous extrusion of the aedeagus by both individuals. This behaviour is accompanied by vigorous, continuous wing fanning performed by both males. This pattern contrasts sharply with male–female encounters, during which only the male exhibits wing fanning.

### Male and female tarsal attachment devices

**Fig. 4:**
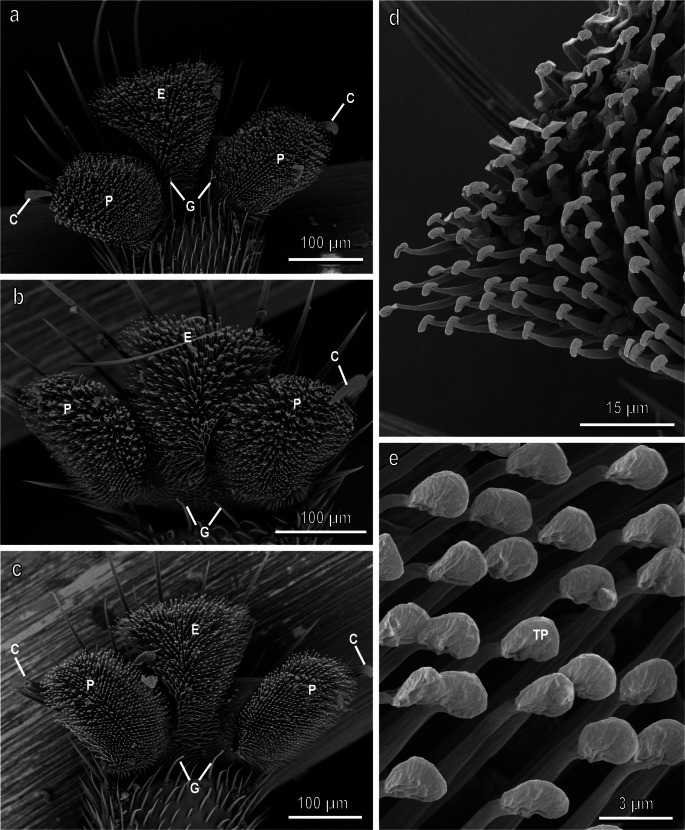
Ventral view of the female pretarsus of *Hermetia illucens* observed in cryo-SEM. Pulvilli (P) and central empodium (E) of the fore legs (**a**), medium legs (**b**) and hind legs (**c**) constituted of a high number of similar tenent setae detailed in (**d**) and (**e**). *C* claws, *G* gustatory sensilla, *TP* terminal plate

**Fig. 5:**
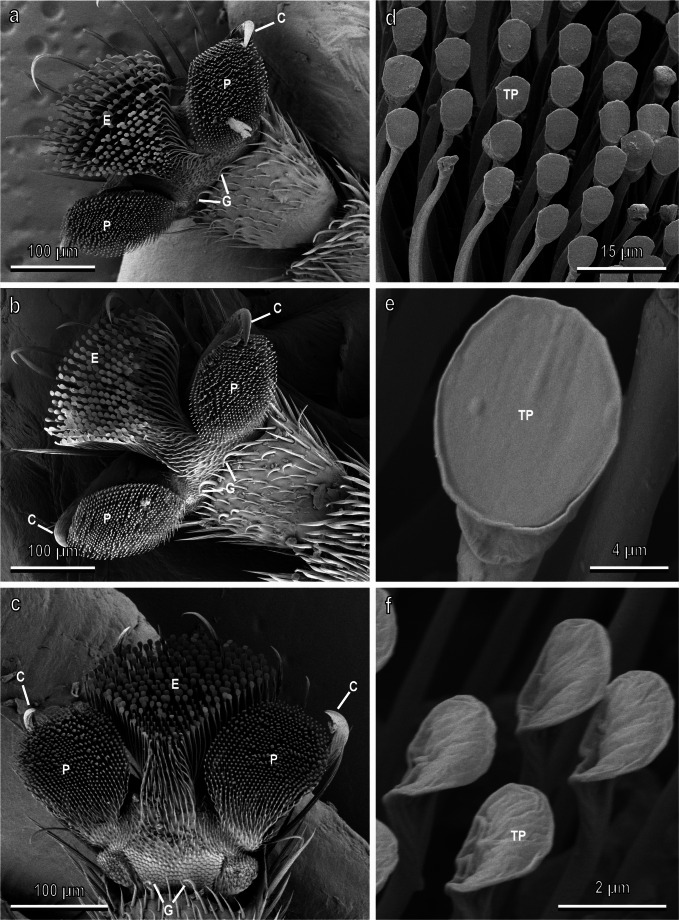
Ventral view of the male pretarsus of *Hermetia illucens* observed in cryo-SEM. Pulvilli (P) and central empodium (E) of the fore legs (**a**), medium legs (**b**) and hind legs (**c**) constituted of a high number of tenent setae whose shape is different in the pulvilli and in the empodium. **d**, **e** Details of the discoidal setae in the empodium; **f** Detail of the spatulate setae in the pulvilli. *C* claws, *G* gustatory sensilla, *TP* terminal plate

**Fig. 6:**
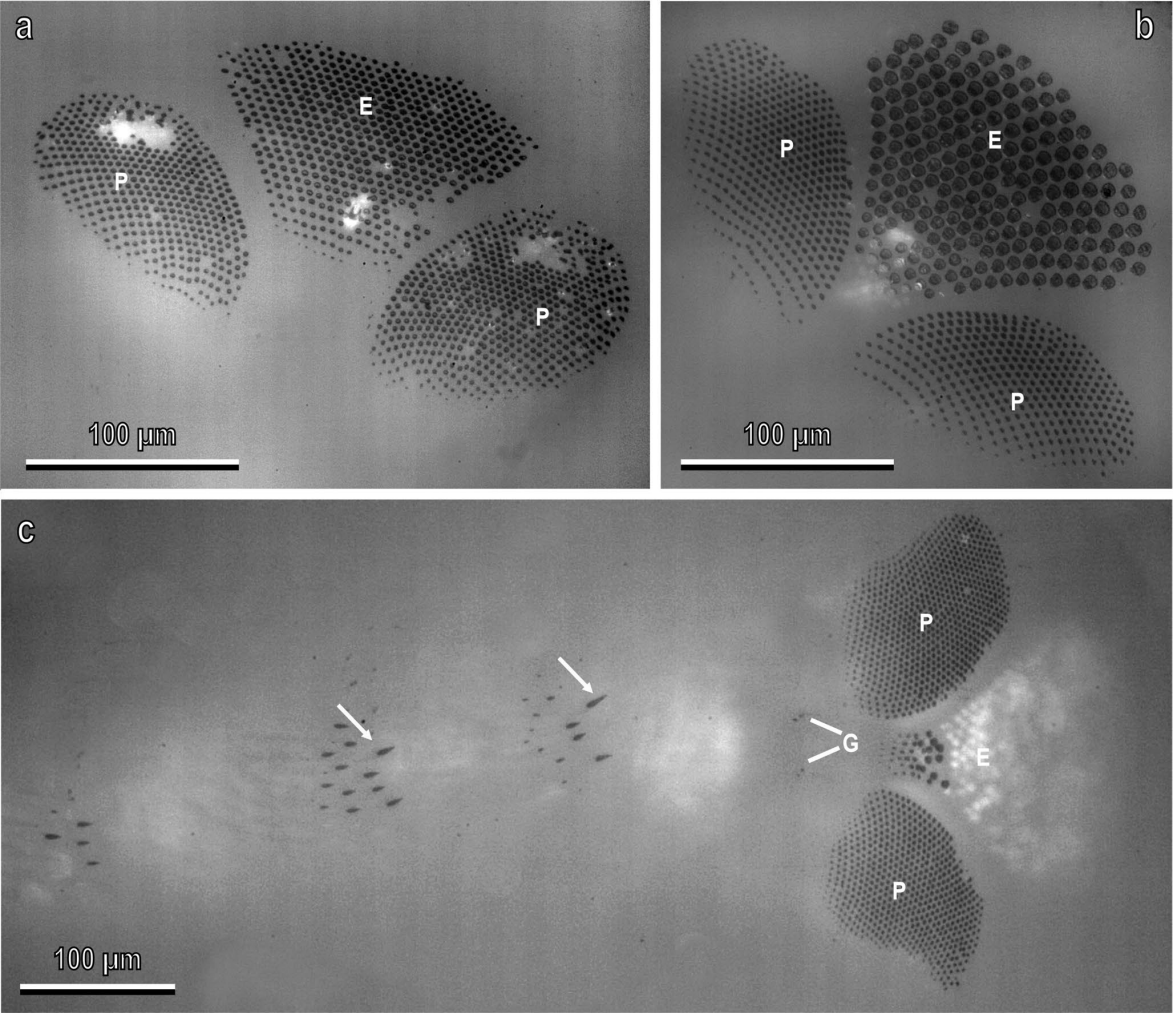
Foreleg contact area of a living individual of *Hermetia illucens* walking on glass, visualised with a combined setup consisting of the reflection-contrast microscope and a high-speed camera. The areas of contact between the attachment structures and the glass appear dark. **a** Contact area of the setae of a female pretarsus with pulvilli (P) and empodium (E) constituted of spatulate setae; **b** Contact area of the setae of a male pretarsus with spatulate setae in pulvilli (P) and discoidal setae in the empodium (E); **c** Contact area of the setae of a male pretarsus. Note the lanceolate setae (arrows) located on the distal portion of each tarsal segment close to gustatory sensilla (G)

Tarsi of adult *H. illucens* consist of five segments (Fig. 3a–c) with the first segment longer than the others (Fig. 3a). The fifth segment bears a pretarsus with the attachment devices constituted of two curved claws located dorsally, two hairy pulvilli and a very well-developed central empodium located ventrally (Figs. 3a, 4 and 5). Long setae emerge from the dorsal side of the last tarsal segment towards the ventral area surrounding the pretarsus (Figs. 4a–c and 5a–c). The two pulvilli and the central empodium are constituted of a high number of tenent setae (Figs. 4 and 5). The correct terminology for these cuticular protuberances should be acanthae since they are not articulated and derive from one epidermal cell, but we call them “tenent setae” according to the terminology introduced for Brachycera by Bauchhenß and Renner (1977), for consistency with the previous literature on fly attachment pads. Each seta is constituted of an unarticulated setal shaft and an adhesive terminal plate (endplate) (Figs. 4d and e and 5d–f). The pulvilli and the empodium are similar in the forelegs, midlegs and hindlegs (Figs. 4a–c and 5a–c), but males and females show sexual dimorphism in the morphology of the endplate of the tenent setae of the empodium. Females have spatulate tenent setae both in the pulvilli and in the empodium (Fig. 4), while males have pulvilli with spatulate setae and empodium with discoidal setae (Fig. 5). The different morphology of the endplate of the tenent setae in the empodium is clearly visible also in male and female tarsal footprints (Fig. 6a, b). Sexual dimorphism can be recognised also in the area of empodium which is significantly wider in males than in females in forelegs (*t* = 3.4; d.f.=21; *P* = 0.003), midlegs (*t* = 4.2; d.f.=21; *P* < 0.001) and hindlegs (*t* = 4.2; d.f.=21; *P* < 0.001) while there is no significant difference in the area of pulvilli between males and females in forelegs (*t* = 0.4; d.f.=21; *P* = 0.701), midlegs (*t* = 0.8; d.f.=21; *P* = 0.437) and hindlegs (*t* = 1; d.f.=21; *P* = 0.329) (Table 1).

Male and female footprints (Fig. 6) reveal that the attachment devices in contact with the substrate during walking are represented not only by the tenent setae of the two pulvilli and of the empodium but also by other setae located on the ventral side of the distal portion of each tarsal segment except the most distal one (Fig. 6c). These tarsal setae are different from those present on the pretarsus: they are lanceolate and similar in males and females (Fig. 3b, c). They are easily distinguishable from uniporous gustatory sensilla located on the distal portion of each tarsal segment (Fig. 3b–d). These sensilla have a curved shape, and their porous apex is bent towards the surface (Fig. 3b–c). The thorax and abdomen of the female are hairy on both the dorsal and ventral surfaces (Fig. 3e). Groups of campaniform sensilla are scattered on the ventral surface of the abdomen (Fig. 3f).

The water contact angle (CA) measured on the dorsal side of the female thorax of *H. illucens* is 139.1 ± 2.1°, thus revealing its hydrophobic properties.

**Table 1:** Area (µm^2^, mean ± SE) of male and female empodium and pulvillus (the right pulvillus was considered) of forelegs, midlegs and hindlegs

Legs	Sex	Empodium area		Pulvillus area	
Foreleg	Male	19,210 ± 811	*	12,398 ± 841	ns
	Female	13,866 ± 1368		11,838 ± 1191	
Midleg	Male	22,210 ± 1003	*	14,140 ± 744	ns
	Female	15,537 ± 1227		13,154 ± 1014	
Hindleg	Male	22,692 ± 863	*	15,666 ± 877	ns
	Female	16,683 ± 1166		14,325 ± 1022	

The asterisk (*) indicates a statistically significant difference (*P* < 0.05), while (ns) indicates that the difference is not statistically significant (*t* test for independent samples)

### Male and female attachment ability

The experiments measuring the friction force exerted by the flies on smooth glass samples with different wettability (hydrophilic glass, CA = 22.9 ± 1.7°; hydrophobic glass, CA = 111.9 ± 0.5°) revealed that in both sexes the friction force was significantly higher on the hydrophilic glass compared to the hydrophobic glass. On hydrophilic glass, females generated stronger forces than males. Considering the force per unit area of contact (mean ± SE), females attached significantly stronger than males to hydrophilic glass (females: 49.72 ± 4.40 mN/mm^2^, males 26.11 ± 1.78 mN/mm^2^, T = 292; *n* = 14; *P* < 0.001).

**Fig. 7:**
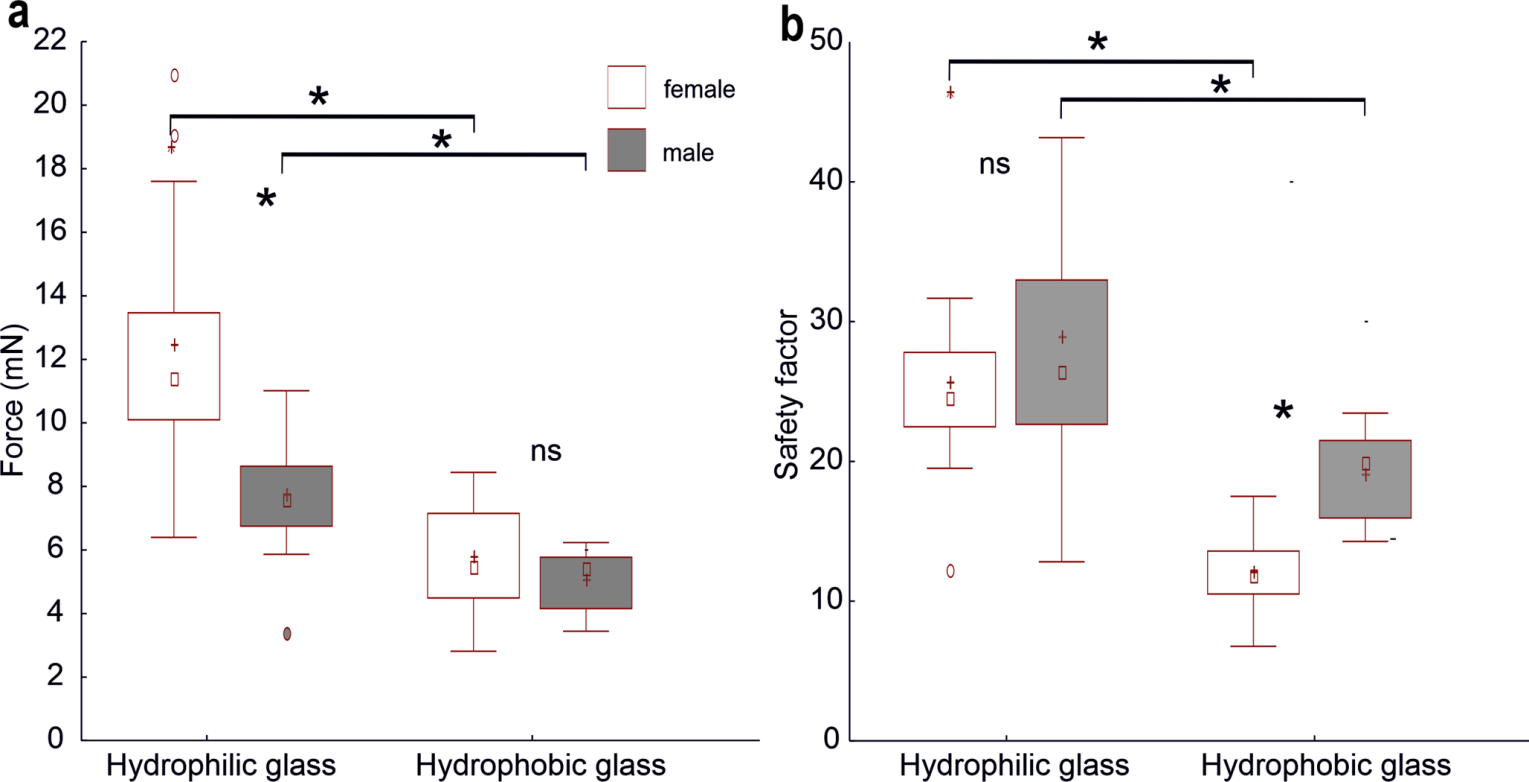
Friction force (**a**) and safety factor (the force divided by insect weight) (**b**) of *Hermetia illucens *females and males on hydrophilic and hydrophobic glass. Boxplots show the interquartile range and the median; whiskers indicate the 1.5 × interquartile range, and “°” indicates outliers. In the comparison between females and males and between the glasses with different wettability, the asterisk (*) means significant difference at *P* < 0.05, and ns means not significantly different (Tukey unequal N HSD post hoc test, One-way ANOVA)

On hydrophobic glass, there was no significant difference in the friction force between the two sexes (sex: F = 10.7; d.f.=1, 20; *P* = 0.004; surface: F = 66.2; d.f.=1, 20; *P* < 0.001; sex x surface: F = 12.5; d.f.=1, 20; *P* = 0.002) (Fig. 7a). When considering the safety factor (friction force divided by insect weight), in both females and males, it was significantly higher on hydrophilic glass compared to hydrophobic glass. On hydrophilic glass, there was no significant difference in the safety factor between the two sexes. Considering the safety factor per unit area of contact (mean ± SE), there was no significant difference in the safety factor on hydrophilic glass between the two sexes (females: 102.11 ± 8.02, males 97.52 ± 9.16, T = 220; *n* = 14; *P* = 0.448).On hydrophobic surfaces, males had higher safety factor than females (sex: F = 7.7; d.f.=1, 20; *P* = 0.012; surface: F = 79.8; d.f.=1, 20; *P* < 0.001; sex x surface: F = 1.8; d.f.=1, 20; *P* = 0.199) (Fig. 7b).

**Fig. 8:**
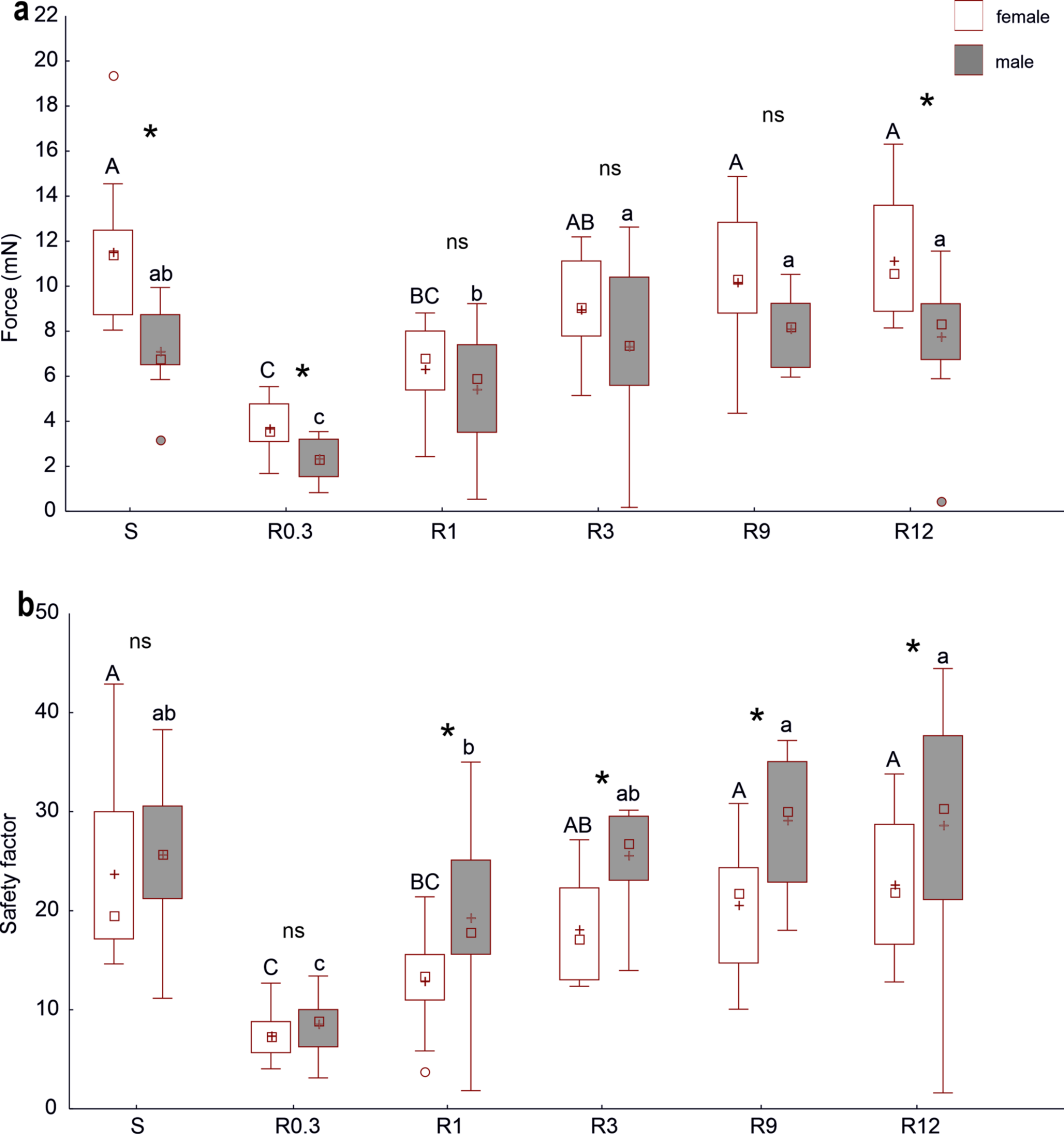
Friction force of *Hermetia illucens* females and males on polishing paper with different asperity sizes (smooth, 0.3, 1, 3, 9, 12). Boxplots show the interquartile range and the medians, whiskers indicate the 1.5 × interquartile range and “°” shows outliers. Boxplots with different upper case letters and lower case letters, respectively, are significantly different at *P* < 0.05 (Tukey unequal N HSD post hoc test, One-way ANOVA). In the comparison between females and males, the asterisk (*) means a significant difference at *P* < 0.05 (*t*-test for independent samples)

The centrifugal experiments on epoxy resin substrates with different roughness (smooth, 0.3, 1, 3, 9, 12 μm) revealed that in females the friction force was significantly higher on smooth surfaces and on surfaces with an asperity size of 9 and 12 μm, intermediate on surfaces with an asperity size of 1 and 3 μm and significantly lower on surfaces with an asperity size of 0.3 μm (F = 20.4; d.f.=5, 50; *P* < 0.001) (Fig. 8a). In males, the friction force was significantly higher on surfaces with an asperity size of 3, 9 and 12 μm, intermediate on surfaces with an asperity size of 1 μm and on smooth surfaces and significantly lower on surfaces with an asperity size of 0.3 μm (F = 23.8; d.f.=5, 49; *P* < 0.001) (Fig. 8a). When comparing female and male friction force, we can observe that female force is higher than male force on smooth surface (*t* = 3.8; d.f.=20; *P* = 0.001), on surface with 0.3 μm asperity size (*t* = 2.9; d.f.=20; *P* = 0.009) and on surface with 12 μm asperity size (*t* = 2.8; d.f.=20; *P* = 0.012) while there is no significant difference in the force between males and females on surfaces with asperity size of 1 μm (*t* = 0.4; d.f.=20; *P* = 0.683), 3 μm (*t* = 0.8; d.f.=19; *P* = 0.409) and 9 μm (*t* = 1.9; d.f.=20; *P* = 0.068) (Fig. 8a).

The safety factor in females was significantly higher on smooth surfaces and on surfaces with an asperity size of 9 and 12 μm, intermediate on surfaces with an asperity size of 1 and 3 μm and significantly lower on surfaces with an asperity size of 0.3 μm (F = 17.9; d.f.=5, 50; *P* < 0.001) (Fig. 8b). In males, the safety factor was significantly higher on surfaces with an asperity size of 9 and 12 μm, intermediate on surfaces with an asperity size of 1, 3 μm and on smooth surfaces and significantly lower on surfaces with an asperity size of 0.3 μm (F = 21.9; d.f.=5, 49; *P* < 0.001) (Fig. 8b).

When comparing female and male safety factors, we observed that male safety factor was higher than the female one on surfaces with 1 μm (*t* = 3.0; d.f.=20; *P* = 0.007), 3 (*t* = 3.4; d.f.=19; *P* = 0.003), 9 μm (*t* = 3.2; d.f.=20; *P* = 0.005) and 12 µm (*t* = 2.4; d.f.=20; *P* = 0.028) asperity size, while there was no significant difference in the safety factor between males and females on the smooth surface (*t* = 0.6; d.f.=20; *P* = 0.580) and on the surface with asperity size of 0.3 μm (*t* = 0.9; d.f.=20; *P* = 0.346) (Fig. 8b).

## Discussion

### Involvement of male tarsi during mating and competition

In agreement with the observations performed in the laboratory on *H. illucens* by Giunti et al. (2018), and in the field on *H. comstocki* (Diptera: Stratiomyidae) by Alcock (1990, 1993), our bioassays reveal that during mating behaviour males follow and grasp flying females, thus showing that in these species flight activity is fundamental to stimulate male courtship. In *H. illucens*, this is probably related to the need to recognise the conspecific at close range of distances using visual cues emitted by dimorphic iridescent wings (Rebora et al. 2024). BSF wings scatter blue colour inducing a strong motivation to mate in BSF males, as it was shown in bioassays (Rebora et al. 2024).

Male grasping in flight, with the hindlegs crossed around the female body, is highlighted here for the first time. This action leads the female to the ground, where copulation occurs. Hind leg crossing behaviour of the male around female body is an interesting behaviour which could have different functions such as to improve mechanical stability during female grasping and also to communicate with the partner to increase female acceptance. This last function has been demonstrated in other Diptera such as Sepsidae where male front legs during mating clamp female wings where numerous sensory organs are located (Eberhard 2002).

Male grasping in flight requires a good attachment ability by male tarsi, where different adhesive setae are located together with gustatory sensilla, as shown in our SEM observations. Tarsi are important during mating for the male tapping behaviour, when the male touches the female abdomen briefly and repeatedly, and also during the male forelegs rubbing on the dorsal body of the female (the male moving his forelegs back and forth on the female body). Tapping and rubbing can function as a form of tactile stimulation of the female body to increase female receptivity and can also be involved in tasting the partner’s cuticular hydrocarbons (CHC) with the gustatory sensilla (potential sexual dimorphism in CHC is an interesting aspect so far not investigated in BSF). Gustatory sensilla are typically present on Diptera tarsi (e.g. Solinas et al. 2001; Stocker 2004) and used in chemical communication during mating in *Drosophila* (Stocker 2004; Han and Kim 2010).

Under rearing conditions, the same behaviour performed by BSF males on female bodies during mating is also performed by males on other males during agonistic encounters, thus leading some authors (Giunti et al. 2018; Julita et al. 2020) to the conclusion that males cannot distinguish between the two sexes. We would exclude this last option, since only males perform wing fanning during both mating and agonistic behaviour. The wing vibration produced by males (so far not investigated in BSF) during this peculiar behaviour could be another important mating signal for the female, as observed in the typical mating dance of *Drosophila* (review in Dickson 2008). From our behavioural observations on BSF under controlled conditions, we can conclude that male attachment ability to the body of conspecifics is very important during mating and agonistic behaviour.

### Sexual dimorphism of adhesive setae

The attachment pads of BSF are represented by two pulvilli, a central developed empodium and the distal area of tarsal segments I-IV. Our observations revealed that male and female attachment devices are dimorphic and represented by three kinds of tenent setae, lanceolate setae located in the distal portion of the tarsal segments (I-IV) of both sexes, spatulate setae located in the female pulvilli and empodium and in the male pulvilli and discoidal setae present only in the male empodium. Sexual dimorphism can also be recognised in the size of empodium which is significantly wider in males than in females in all the legs in spite of the different sizes of males and females, with females being bigger than males.

Diptera show typically hairy pads (Beutel and Gorb 2001). These pads are represented mainly by pulvilli and empodium (this last is not developed in all species), with few species showing, in addition to pulvilli and empodium, adhesive setae on the ventral surface of the tarsal segments (Friedemann et al. 2015). The presence of a well-developed pad-like empodium like that observed in *H. illucens* is a potential autapomorphy of Diptera, and in the ‘higher’ Brachycera there is a trend towards reduction of this pad (Friedemann et al. 2015). Its presence in *H. illucens* is in agreement with the basal position of Stratiomyidae among Brachycera.

Different species belonging to higher Diptera, such as Calliphoridae, Muscidae and Syrphidae with hairy pulvilli and reduced empodium, have been used as model insects to shed light on basic principles underlining the mechanism of insect attachment ability (Bauchhenß and Renner 1977; Bauchhenß 1979; Walker et al. 1985; Gorb et al. 2001; Niederegger et al. 2002; Niederegger and Gorb 2003; Gorb et al. 2012; Peisker and Gorb 2012; Peisker et al. 2014). Similar adhesive pads of some Diptera species belonging to Tephritidae and representing important pest species, such as *Bactrocera oleae* (Rossi) and *Ceratitis capitata* Wiedemann, have been studied in detail in relation to the attachment ability to host plant surfaces with different morphological features (Rebora et al. 2020; Salerno et al. 2020). In all these species, tenent setae have a rigid shaft and a highly flexible terminal plate with special exocuticle (Salerno et al. 2020) and a high amount of resilin (Peisker et al. 2013; Gorb and Filippov 2014) which helps the seta endplate to deform and replicate the surface profile, increasing the pad adaptability to different kinds of surfaces (Gorb et al. 2000; Eimüller et al. 2008; Scholz et al. 2008). The contact with the surface by bending the tips in the distal direction is mediated by fluids (Walker et al. 1985; Gorb et al. 2012; Peisker and Gorb 2012).

In the overview on the attachment structures in lower Diptera by Friedemann et al. (2014), the tenent setae have a terminal plate with various shapes in the different analysed taxa. It is almost always distinctly broadened, but the shape varies. Often in fly pulvilli, distal “tenent setae” have a circular terminal plate while the proximal “tenent setae” have a narrower terminal plate (e.g. in *C. capitata*, Salerno et al. 2020). The terminal plate can be round, triangular or tapered (Friedemann et al. 2014).

The sexual dimorphism of adhesive pads in Diptera concerns some differences in the area of male and female pulvilli, such as in *Calliphora erythrocephala* (L.) (Gorb 2001), but nothing similar to that observed in *H. illucens* in the present study, with discoidal setae visible only in male empodium, has been described so far in flies. Such a situation resembles that described in many beetle species, where males and females exhibit clear structural differences in the setae of their tarsal adhesive pads (Stork 1980; Voigt et al. 2008, 2017; Gorb et al. 2010; Heepe et al. 2018; Saitta et al. 2025). Females usually bear needle-like or spatulate setae, while males, in addition, possess highly specialized disco-setae, characterized by a rigid shaft (Heepe et al. 2018) ending in a rounded to oval plate (Stork 1980), similar to those described in BSF. In Coleoptera, these specialized setae are believed to be able to provide strong adhesive performance on smooth surfaces (Gorb et al. 2007), such as female elytra during mating (Alcock 2006; Matsumura et al. 2023). In line with this, higher adhesive forces have been measured in males on smooth surfaces compared to females in some beetle species (Coleoptera: Coccinellidae) (Voigt et al. 2017; Gorb et al. 2019).

In the BSF, the situation is different: in our centrifugal experiments, measuring male and female attachment ability to epoxy resin substrates with different roughness (smooth, 0.3, 1, 3, 9, 12 μm), we could not observe higher attachment ability of males compared to females on smooth surfaces. The safety factor (force divided by the insect weight, since females are bigger than males, it is better to use this value) on smooth hydrophilic surfaces is similar in the two sexes, even considering the mean safety factor per unit area of contact and the performance on the different surfaces is comparable to that observed in the Mediterranean fruit fly *C. capitata* (Salerno et al. 2020), which shows only hairy pulvilli and not a hairy empodium and no discoidal setae.

Further morphological and behavioural studies testing the attachment ability on surfaces with different features on fly species closely related to *H. illucens* are necessary to clarify the role of this remarkable sexual dimorphism in the BSF.

### Attachment ability on hydrophilic and hydrophobic surfaces

As reported above, BSF males require a good attachment ability to the body of conspecifics during mating behaviour and their body surface (thorax, abdomen) is not smooth, but rather rich of setae and also it is hydrophobic with a CA of 139.1 ± 2.1°. In this regard, it is interesting to compare the BSF attachment ability on smooth surfaces with different wettability (hydrophilic glass, CA = 22.9 ± 1.7° and hydrophobic glass, CA = 111.9 ± 0.5°). Both males and females perform better on hydrophilic than on hydrophobic surfaces as highlighted in many other insect species belonging to Diptera (Rebora et al. 2020; Salerno et al. 2020), Hemiptera (Friedemann et al. 2015; Salerno et al. 2017), Hymenoptera (Voigt and Gorb 2012; Stark and Yanoviak 2018) and Coleoptera (Gorb and Gorb 2009, 2020; Lüken et al. 2009; Gorb et al. 2010; Hosoda and Gorb 2012; Zurek et al. 2015; Salerno et al. 2020, 2022) tested so far. This is probably due to the low affinity of the insect adhesive fluid to the hydrophobic substrates due to its chemical composition, which in flies is assumed to be a mainly water-based microemulsion with low lipid content (Peisker and Gorb 2012).

When we compare the different performance of BSF males and females on hydrophilic and hydrophobic surfaces, it is interesting to note that male attachment ability is less affected by hydrophobic surfaces compared with females and the male safety factor on hydrophobic surfaces is higher than that of females. These results could be related to some adaptation present in males to enhance adhesion during mating. Such adaptation could be similar to what is observed in some parasitoid wasps, where sexual dimorphism in the attachment ability has been observed, with females’ attachment systems tuned to match the wettability of the host surface (Rebora et al. 2022; Salerno et al. 2024). As reported for Hymenoptera parasitoids (Salerno et al. 2024), a possible explanation for the different ability of males and females to adhere to surfaces with different wettability may be the different chemical composition of the tarsal fluid in the two sexes. Further studies are necessary to clarify this aspect.

### Attachment ability to artificial surfaces with different roughness

The performance of BSF males and females on artificial surfaces with different roughness is similar to that observed in many other insect species with smooth and hairy adhesive pads belonging to different orders (Peressadko and Gorb 2004; Voigt et al. 2008; Bullock and Federle 2011; Zurek et al. 2017; Salerno et al. 2017, 2018) and showing higher attachment ability on smooth surfaces (S) and rough surfaces (3, 9, 12 μm asperity size) and lower attachment to microrough surfaces (0.3, 1 μm asperity size). The typical low attachment performance of insects on these last surfaces, confirmed also in the BSF, is due to tarsal adhesive pads, which can generate mainly contact on smooth substrate or smooth islands of substrates with large roughness, whereas a reduction of the contact area results from small surface irregularities (Gorb 2001; Peressadko and Gorb 2004; Voigt et al. 2008; Zhou et al. 2014; Zurek et al. 2017; Kovalev et al. 2018).

On surfaces with higher asperity dimensions (3, 9, 12 μm), the BSF can adhere not only to smooth islands within rough substrates with the terminal plate of their tenent setae but also interlock using their claws with the substrate asperities larger than the claw tip diameter (Dai et al. 2002; Song et al. 2016). Such information, together with the data about adult performance on different artificial surfaces with different wettability collected in the present study, can be useful to develop artificial substrates in rearing conditions aimed to increase or decrease adult attachment of the BSF, in order to improve mass raring techniques.

## Conclusions

For commercially important insects (like BSF), most research focuses on larval growth (for feed/fertilizer), neglecting adult stage and its reproductive behaviour. However, to realise an efficient mating process yielding fertile eggs, in order to produce a steady supply of neonate larvae, knowledge regarding adult reproductive behaviour – e.g. criteria and sensory cues involved in intra- and intersexual selection, interaction between partners during mating and female oviposition behaviour – are necessary (Lemke et al. 2023). In this regard, the present study sheds light on adult attachment ability, adult sexual dimorphism and interaction between conspecifics during mating and competition under rearing conditions. Further studies on these aspects, deepening the knowledge on adult behaviour in the wild and under rearing conditions, can be important to enhance performance of BSF mass-rearing facilities.

## Data Availability

The data are available upon request to the authors.

## References

[CR1] Alcock J (1990) A large male competitive advantage in a lekking fly, *Hermetia comstocki* Williston (Diptera: Stratiomyidae). Psyche 97:267–279. 10.1155/1990/72328

[CR2] Alcock J (1993) The effects of male body size on territorial and mating success in the landmark-defending fly *Hermetia comstocki* (Stratiomyidae). Ecol Entomol 18:1–6. 10.1111/j.1365-2311.1993.tb01073.x

[CR3] Alcock J (2006) Animal Behavior: An Evolutionary Approach 8th ed. Elsevier/Sinauer. ISBN: 9780878930050

[CR5] Bauchhenß E (1979) Die Pulvillen von *Calliphora erythrocephala* Meig. (Diptera, Brachycera) als Adhäsionsorgane. Zoomorphology 93:99–123. 10.1007/BF00994125

[CR4] Bauchhenß E, Renner M (1977) Pulvillus of *Calliphora erythrocephala* Meig (Diptera. Int J Insect Morphol Embryol 6:225–227. 10.1016/0020-7322(77)90010-1. Calliphoridae

[CR6] Beutel RG, Gorb SN (2001) Ultrastructure of attachment specializations of hexapods (Arthropoda): evolutionary patterns inferred from a revised ordinal phylogeny. J Zool Syst Evol Res 39(4):177–207. 10.1046/j.1439-0469.2001.00155.x

[CR7] Billeter JC, Levine JD (2013) Who Is He and What Is He to You? Recognition in *Drosophila melanogaster*. Curr Opin Neurobiol 23:17–23. 10.1016/j.conb.2012.08.009 23010098 10.1016/j.conb.2012.08.009

[CR9] Bruno D, Manas F, Bonelli M, Gold M, Marzari M, Roma D, Valoroso MC, Montali A, Guillaume JB, Rebora M, Bressac C, Herman N, Caccia S, Casartelli M, Tettamanti G (2025) BugBook: Life cycle, reproduction, and morphofunctional characterisation of the gut, fat body, and haemocytes in the black soldier fly. J Insects Food Feed 11:289–316. 10.1163/23524588-20250002

[CR8] Bullock JMR, Federle W (2011) The effect of surface roughness on claw and adhesive hair performance in the dock beetle *Gastrophysa viridula*. Insect Sci 18:298–304. 10.1111/j.1744-7917.2010.01369.x

[CR10] Dai Z, Gorb SN, Schwarz U (2002) Roughness-dependent friction force of the tarsal claw system in the beetle *Pachnoda marginata* (Coleoptera, Scarabaeidae). J Exp Biol 205:2479–2488. 10.1242/jeb.205.16.2479 12124371 10.1242/jeb.205.16.2479

[CR11] Dickson BJ (2008) Wired for sex: the neurobiology of Drosophila mating decisions. Science 322:904–909. 10.1126/science.1159276 18988843 10.1126/science.1159276

[CR12] Eberhard WG (2002) Physical restraint or stimulation? The function(s) of the modified front legs of male *Archisepsis diversiformis* (Diptera: Sepsidae). J Insect Behav 15(6):831–850. 10.1023/A:1021161915227

[CR13] Eimuller T, Guttmann P, Gorb SN (2008) Terminal contact elements of insect attachment devices studied by transmission X-ray microscopy. J Exp Biol 211(12):1958–1963. 10.1242/jeb.014308 18515726 10.1242/jeb.014308

[CR14] Friedemann K, Schneeberg K, Beutel RG (2014) Fly on the wall – attachment structures in lower Diptera. Syst Entomol 39:460–473. 10.1111/syen.12064

[CR15] Friedemann K, Kunert G, Gorb E, Gorb SN, Beutel RG (2015) Attachment forces of pea aphids (*Acyrthosiphon pisum*) on different legume species. Ecol Entomol 40:732–740. 10.1111/een.12249

[CR16] Giunti G, Campolo O, Laudani F, Palmeri V (2018) Male courtship behaviour and potential for female mate choice in the black soldier fly *Hermetia illucens* L. (Diptera, Stratiomyidae). Entomol Gen 38:29–46. 10.1127/entomologia/2018/0657

[CR17] Gorb SN (2001) Attachment devices of insect cuticle. Kluwer Academic

[CR18] Gorb SN (2007) Visualisation of native surfaces by two-step molding. Microsc Today 15:44–46. 10.1017/S1551929500051038

[CR19] Gorb SN, Filippov AE (2014) Fibrillar adhesion with no clusterisation: functional significance of material gradient along adhesive setae of insects. Beilstein J Nanotechnol 5(1):837–845. 10.3762/bjnano.5.95 24991520 10.3762/bjnano.5.95 PMC4077360

[CR20] Gorb E, Gorb S (2009) Effects of surface topography and chemistry of *Rumex obtusifolius* leaves on the attachment of the beetle *Gastrophysa viridula*. Entomol Exp Appl 130:222–228. 10.1111/j.1570-7458.2008.00806.x

[CR21] Gorb EV, Gorb SN (2020) Attachment ability of females and males of the ladybird beetle *Cryptolaemus montrouzieri* to different artificial surfaces. J Insect Physiol 121:104011. 10.1016/j.jinsphys.2019.104011 31904387 10.1016/j.jinsphys.2019.104011

[CR23] Gorb SN, Jiao Y, Scherge M (2000) Ultrastructural architecture and mechanical properties of attachment pads in *Tettigonia viridissima* (Orthoptera Tettigoniidae). J Comp Physiol A 186:821–831. 10.1007/s003590000135 11085636 10.1007/s003590000135

[CR22] Gorb S, Gorb E, Kastner V (2001) Scale effects on the attachment pads and friction forces in syrphid flies (Diptera, Syrphidae). J Exp Biol 204:1421–1431. 10.1242/jeb.204.8.1421 11273804 10.1242/jeb.204.8.1421

[CR27] Gorb SN, Varenberg M, Peressadko A, Tuma J (2007) Biomimetic mushroom-shaped fibrillar adhesive microstructure. J R Soc Interface 4:271–275. 10.1098/rsif.2006.0164 17251156 10.1098/rsif.2006.0164 PMC2359835

[CR25] Gorb EV, Hosoda N, Miksch C, Gorb SN (2010) Slippery pores: antiadhesive effect of nanoporous substrates on the beetle attachment system. J R Soc Interface 7:1571–1579. 10.1098/rsif.2010.0081 20427333 10.1098/rsif.2010.0081 PMC2988254

[CR24] Gorb SN, Schuppert J, Walther P, Schwarz H (2012) Contact behaviour of setal tips in the hairy attachment system of the fly *Calliphora vicina* (Diptera, Calliphoridae): a cryo-SEM approach. Zoology 115(3):142–150. 10.1016/j.zool.2011.10.006 22554589 10.1016/j.zool.2011.10.006

[CR26] Gorb EV, Lemke W, Gorb SN (2019) Porous substrate affects a subsequent attachment ability of the beetle *Harmonia axyridis* (Coleoptera, Coccinellidae). J R Soc Interface 16:20180696. 10.1098/rsif.2018.0696 30958175 10.1098/rsif.2018.0696 PMC6364653

[CR28] Han KA, Kim YC (2010) Courtship behavior: the right touch stimulates the proper song. Curr Biol 20. 10.1016/j.cub.2009.11.060. R25-R28 10.1016/j.cub.2009.11.060 20152141

[CR29] Heepe L, Höft S, Michels J, Gorb SN (2018) Material gradients in fibrillar insect attachment systems: the role of joint-like elements. Soft Matter 14:7026–7033. 10.1039/C8SM01151F 30109340 10.1039/c8sm01151f

[CR30] Hosoda N, Gorb SN (2012) Underwater locomotion in a terrestrial beetle: combination of surface de-wetting and capillary forces. Proc R Soc B 279:4236–4242. 10.1098/rspb.2012.1297 10.1098/rspb.2012.1297 PMC3441071 22874756

[CR31] Julita U, Fitri LL, Putra RE, Permana AD (2020) Mating success and reproductive behavior of black soldier fly *Hermetia illucens* L. (Diptera, Stratiomyidae) in tropics. J Entomol 17:117–127. 10.3923/je.2020.117.127

[CR32] Kovalev A, Filippov AE, Gorb SN (2018) Critical roughness in animal hairy adhesive pads: A numerical modeling approach. Bioinspir Biomim 13:066003. 10.1088/1748-3190/aadd66 30156566 10.1088/1748-3190/aadd66

[CR33] Lemke NB (2025) All about lekking: A comprehensive examination of lekking and the lek mating system in the Black Soldier Fly, *Hermetia illucens*. Preprints 2025:2025010515. 10.20944/preprints202501.0515.v1

[CR34] Lemke NB, Dickerson AJ, Tomberlin JK (2023) No neonates without adults: A review of adult black soldier fly biology. BioEssays 45:e2200162. 10.1002/bies.202200162 36382549 10.1002/bies.202200162

[CR35] Lüken D, Voigt D, Gorb SN, Zebitz CPW (2009) Die Tarsenmorphologie und die Haftfähigkeit des Schwarzen Batatenkäfers *Cylas puncticollis* (Boheman) auf glatten Oberflächen mit unterschiedlichen physiko-chemischen Eigenschaften. Mitt Dtsch Ges Allg Angew Entomol 17:109–113

[CR36] Matsumura Y, Gorb EV, Gorb SN (2023) The tight attachment achieved by the male discoidal setae is possibly a counter-adaptation to the grease layer on female integument surfaces in green dock beetles. J R Soc Interface 20:20230324. 10.1098/rsif.2023.0324 37582406 10.1098/rsif.2023.0324 PMC10427193

[CR37] Meneguz M, Miranda CD, Cammack JA, Tomberlin JK (2023) Adult behaviour as the next frontier for optimising industrial production of the black soldier fly *Hermetia illucens* (L.) (Diptera: Stratiomyidae). J Insects Food Feed 9:399–414. 10.3920/JIFF2022.0055

[CR38] Mohan K, Sathishkumar P, Rajan DK, Rajarajeswaran J, Ganesan AR (2023) Black soldier fly (*Hermetia illucens*) larvae as potential feedstock for the biodiesel production: Recent advances and challenges. Sci Total Environ 859:160235. 10.1016/j.scitotenv.2022.160235 36402342 10.1016/j.scitotenv.2022.160235

[CR40] Niederegger S, Gorb S (2003) Tarsal movements in flies during leg attachment and detachment on a smooth substrate. J Insect Physiol 49(6):611–620. 10.1016/S0022-1910(03)00048-9 12804721 10.1016/s0022-1910(03)00048-9

[CR39] Niederegger S, Gorb SN, Jiao Y (2002) Contact behaviour of tenent setae in attachment pads of the blowfly *Calliphora vicina* (Diptera, Calliphoridae). J Comp Physiol A 187:961–970. 10.1007/s00359-001-0265-7 10.1007/s00359-001-0265-7 11913814

[CR41] Peisker H, Gorb SN (2012) Evaporation dynamics of tarsal liquid footprints in flies (*Calliphora vicina*) and beetles (*Coccinella septempunctata*). J Exp Biol 215:1266–1271. 10.1242/jeb.065722 22442363 10.1242/jeb.065722

[CR43] Peisker H, Michels J, Gorb SN (2013) Evidence for a material gradient in the adhesive tarsal setae of the ladybird beetle *Coccinella septempunctata*. Nat commun 4(1):1661. 10.1038/ncomms2576 23552076 10.1038/ncomms2576

[CR42] Peisker H, Heepe L, Kovalev AE, Gorb SN (2014) Comparative study of the fluid viscosity in tarsal hairy attachment systems of flies and beetles. J R Soc Interface 11(99):20140752. 10.1098/rsif.2014.0752 25142527 10.1098/rsif.2014.0752 PMC4233759

[CR44] Peressadko AG, Gorb SN (2004) Surface profile and friction force generated by insects. In: Boblan I, Bannasch R (eds) First International Industrial Conference Bionik, 2004. VDI Verlag, Düsseldorf, pp 257–261

[CR45] Piersanti S, Rebora M, Marri GC, Salerno G (2024) Antennal olfactory responses in the black soldier fly *Hermetia illucens*. J Insect Physiol 166:104618. 10.1016/j.jinsphys.2024.104722 10.1016/j.jinsphys.2024.104722 39542085

[CR46] Piersanti S, Salerno G, Gentile M, Paccagnini E, Rebora M (2026) Ultrastructure of the black soldier fly antennal sensilla. Cell Tissue Res 403:2. 10.1007/s00441-025-04041-6 41535515 10.1007/s00441-025-04041-6

[CR47] Rebora M, Salerno G, Piersanti S, Gorb E, Gorb S (2020) Role of fruit epicuticular waxes in preventing *Bactrocera oleae* attachment. Insects 11:189. 10.3390/insects11030189 32192070 10.3390/insects11030189 PMC7142657

[CR48] Rebora M, Salerno G, Piersanti S, Saitta V, Gorb E, Gorb SN (2022) Mechanical interaction of the egg parasitoid *Anastatus bifasciatus* with artificial substrates and its host egg. Front Mech Eng 8:966429. 10.3389/fmech.2022.966429

[CR49] Rebora M, Piersanti S, Romani A, Kovalev A, Gorb S, Salerno G (2024) Sexual dimorphism in the structural colours of the wings of the black soldier fly (BSF) *Hermetia illucens* (Diptera: Stratiomyidae). Sci Rep 14(1):19655. 10.1038/s41598-024-70684-0 39179757 10.1038/s41598-024-70684-0 PMC11343838

[CR50] Saitta V, Rebora M, Piersanti S, Carboni Marri G, Masini P, Gorb E, Iacovone A, Salerno G, Gorb S (2025) Sexual Dimorphism of Tarsal Attachment Devices and Their Relation to Mating in Coccinellidae. J Morphol 286(4):e70041. 10.1002/jmor.70041 40181652 10.1002/jmor.70041 PMC11969132

[CR51] Salerno G, Rebora M, Gorb EV, Kovalev A, Gorb SN (2017) Attachment ability of the southern green stink bug *Nezara viridula*. J Comp Physiol A 203:1–11. 10.1007/s00359-017-1177-5 10.1007/s00359-017-1177-5 28488067

[CR52] Salerno G, Rebora M, Kovalev A, Gorb EV, Gorb SN (2018) Contribution of different tarsal attachment devices to the overall attachment ability of the stink bug *Nezara viridula*. J Comp Physiol A 204:627–638. 10.1007/s00359-018-1266-0 10.1007/s00359-018-1266-0 29777384

[CR53] Salerno G, Rebora M, Piersanti S, Gorb E, Gorb S (2020) Mechanical ecology of fruit-insect interaction in the adult Mediterranean fruit fly *Ceratitis capitata*. Zoology 139:125748. 10.1016/j.zool.2020.125748 32078916 10.1016/j.zool.2020.125748

[CR54] Salerno G, Rebora M, Piersanti S, Büscher TH, Gorb EV, Gorb SN (2022) Oviposition site selection and attachment ability of *Propylea quatuordecimpunctata* and *Harmonia axyridis*. Physiol Entomol 47:20–37. 10.1111/phen.12368

[CR55] Salerno G, Rebora M, Piersanti S, Gorb E, Gorb S (2024) Parasitoid attachment ability and the host surface wettability. Zoology 165:126181. 10.1016/j.zool.2024.126181 38833995 10.1016/j.zool.2024.126181

[CR56] Schmitt E, de Vries W (2020) Potential benefits of using *Hermetia illucens* frass as a soil amendment on food production. Curr Opin Green Sustain Chem 25:100335. 10.1016/j.cogsc.2020.100335

[CR57] Scholz I, Baumgarnter W, Federle W (2008) Micromechanics of smooth adhesive organs in stick insects: pads are mechanically anisotropic and softer towards the adhesive surface. J Comp Physiol A 194:373–384. 10.1007/s00359-008-0314-6 10.1007/s00359-008-0314-6 18219488

[CR58] Scieuzo C, Nardiello M, Farina D, Scala A, Cammack JA, Tomberlin JK, Vogel H, Salvia R, Persaud K, Falabella P (2021) *Hermetia illucens* odorant binding proteins and their interactions with volatile organic compounds. Insects 12:814. 10.3390/insects12090814 34564254 10.3390/insects12090814 PMC8469849

[CR59] Solinas M, Rebora M, De Cristofaro A (2001) Functional morphology of *Bactrocera oleae* Gmel.(Diptera: Tephritidae) tarsal chemosensilla involved in interactions with the host-plant. Entomologica. 35:103–123. 10.15162/0425-1016/734

[CR60] Song Y, Dai Z, Wang Z, Ji A, Gorb SN (2016) The synergy between the insect-inspired claws and adhesive pads increases the attachment ability. Sci Rep 6:26219. 10.1038/srep26219 27198650 10.1038/srep26219 PMC4873747

[CR61] Stark AY, Yanoviak SP (2018) Adhesion and running speed of a tropical arboreal ant (*Cephalotes atratus*) on wet substrates. R Soc Open Sci 5:171151 10.1098/rsos.181540 10.1098/rsos.181540 PMC6281928 30564427

[CR63] Stocker RF (2004) Taste perception: Drosophila – a model of good taste. Curr Biol 14:R560–R561. 10.1016/j.cub.2004.07.011 15268874 10.1016/j.cub.2004.07.011

[CR64] Stork NE (1980) Experimental analysis of adhesion of *Chrysolina polita* (Chrysomelidae: Coleoptera) on a variety of surfaces. J Exp Biol 88(1):91–108. 10.1242/jeb.88.1.91

[CR65] Tomberlin JK, Sheppard DC (2001) Lekking behavior of the black soldier fly (Diptera: Stratiomyidae). Fla Entomol 84:729–736. 10.2307/3496413

[CR66] Tomberlin JK, Van Huis A (2020) Black soldier fly from pest to ‘crown jewel’ of the insects as feed industry. J Insects Food Feed 6:1–4. 10.3920/JIFF2020.0003

[CR67] Voigt D, Gorb SN (2012) Attachment ability of sawfly larvae to smooth surfaces. Arthropod Struct Dev 41:145–153. 10.1016/j.asd.2011.10.001 22289716 10.1016/j.asd.2011.10.001

[CR68] Voigt D, Schuppert JM, Dattinger S, Gorb SN (2008) Sexual dimorphism in the attachment ability of the Colorado potato beetle *Leptinotarsa decemlineata* (Coleoptera: Chrysomelidae) to rough substrates. J Insect Physiol 54:765-776. 10.1016/j.jinsphys.2008.02.006 10.1016/j.jinsphys.2008.02.006 18387627

[CR70] Voigt D, Tsipenyuk A, Varenberg M (2017) How tight are beetle hugs? Attachment in mating leaf beetles. R Soc Open Sci 4:171108. 10.1098/rsos.171108 28989792 10.1098/rsos.171108 PMC5627132

[CR71] Walker G, Yule AB, Ratcliffe J (1985) The adhesive organ of the blowfly, *Calliphora vomitoria*: a functional approach. J Zool 205:297–307. 10.1111/j.1469-7998.1985.tb03536.x

[CR72] Wang YS, Shelomi M (2017) Review of black soldier fly (*Hermetia illucens*) as animal feed and human food. Foods 6:91. 10.3390/foods6100091 29057841 10.3390/foods6100091 PMC5664030

[CR73] Zhou Y, Robinson A, Steiner U, Federle W (2014) Insect adhesion on rough surfaces: analysis of adhesive contact of smooth and hairy pads. J R Soc Interface 11:20140499. 10.1098/rsif.2014.0499 24990289 10.1098/rsif.2014.0499 PMC4233698

[CR74] Zurek DB, Gorb SN, Voigt D (2015) Locomotion and attachment of leaf beetle larvae *Gastrophysa viridula* (Coleoptera, Chrysomelidae). Interface Focus 5(1):20140055. 10.1098/rsfs.2014.0055 25657837 10.1098/rsfs.2014.0055 PMC4275872

[CR75] Zurek DB, Gorb SN, Voigt D (2017) Changes in tarsal morphology and attachment ability to rough surfaces during ontogenesis in *Gastrophysa viridula*. Arthropod Struct Dev 46:130–137. 10.1016/j.asd.2016.09.006 27664782 10.1016/j.asd.2016.09.006

